# Implementing Routine HIV Testing: The Role of State Law

**DOI:** 10.1371/journal.pone.0001005

**Published:** 2007-10-10

**Authors:** Leslie E. Wolf, Alexis Donoghoe, Tim Lane

**Affiliations:** 1 Program in Medical Ethics, Center for AIDS Prevention Studies, University of California at San Francisco, San Francisco, California, United States of America; 2 University of San Francisco School of Law, San Francisco, California, United States of America; 3 Center for AIDS Prevention Studies, University of California at San Francisco, San Francisco, California, United States of America; Instituto de Pesquisa Clinica Evandro Chagas-FIOCRUZ, Brazil

## Abstract

In September 2006, the Centers for Disease Control and Prevention (CDC) recommended routine HIV testing for all Americans aged 13–64, which would eliminate requirements for written consent and pretest counseling as previously required. However, this approach may conflict with state requirements concerning pretest counseling and informed consent for HIV testing. Our survey of state HIV testing laws demonstrates that the majority of states have HIV testing requirements that are inconsistent with the CDC's recommendations. Moreover, states that have recently amended their laws have not eased the requirements for pretest counseling and informed consent. The reasons for the persistence of these legal requirements must be understood to effect policy changes to increase HIV testing.

## Introduction

In September 2006, the Centers for Disease Control and Prevention (CDC) recommended routine HIV testing for all Americans between 13 and 64. According to the CDC's recommendations, routine HIV testing means that all patients would be told that HIV testing is a routine part of care and they will be tested unless they decline [1]. The CDC specifically recommends eliminating requirements for specific consent to HIV testing and pretest counseling, a significant departure from previous HIV testing policy [1], [2].

The CDC's new recommendations are aimed at increasing the number of people who know their HIV status to reduce transmission [2]–[4]. People who are unaware they are HIV-infected account for an estimated 20,000 new HIV infections annually [5]. People often do not test for HIV because they do not perceive themselves at risk of infection [6]–[8]. Although patients are more likely to test when their physicians suggest it [6], [9], many physicians do not do so because pretest counseling takes considerable time and discussing sexual and drug behaviors that risk transmission may be uncomfortable [10], [11], [12]. It is hoped that physicians will offer HIV testing more often–and, thus, more people will test–if it is viewed as a routine part of care and only notification is required [1]. Moreover, because there is no assessment of sexual or drug-using risk, routine testing may reduce the stigma of HIV testing [9]. Increasing HIV testing may reduce transmission rates because people change their behaviors when they know they are HIV-infected and because appropriate HIV treatment can reduce viral load and thus decrease infectivity [1], [10], [13]. It can also reduce morbidity by helping people who are HIV-infected get appropriate treatment [10], [13]. The CDC recommends repeating testing at least annually for those at high risk for HIV and as needed based on clinical judgment for others [1]. Although there are costs to expanded testing, studies demonstrate that routine HIV testing is a cost-effective means of achieving these public health goals, even in low-prevalence populations [5], [14].

Although there is widespread support for broader HIV testing, HIV/AIDS advocates have expressed concerns about removing protections such as written informed consent and pretest counseling [15]–[19]. They argue that these processes are essential to helping people understand the potential negative psychosocial consequences of testing, particularly if results are positive, such as stigma and discrimination [19]–[21]. Moreover, pretest counseling is important to HIV prevention because it educates people about reducing risk of infection, regardless of HIV-status [19]–[22]. In addition, written consent for HIV testing protects patients and physicians, while promoting patient information and awareness [23].

The CDC and commentators have acknowledged that states' laws might limit implementation of routine testing [1], [4]; however, none of the discussions have seriously addressed the extent of these potential legal barriers. Because there is a national debate about how to implement the CDC's recommendations [24]–[26] and some organizations already have begun implementation [17], [27], it is essential to understand how laws may affect these efforts. We previously reported results of a survey of HIV testing statutes and regulations in the 50 states and the District of Columbia in connection with recommendations for routine HIV testing of pregnant women that documented legal barriers to implementation of those recommendations [28]. For this article, we updated our previous legal research to determine whether states have changed their HIV testing laws to make it feasible to implement routine testing. In particular, we looked at whether states had laws or regulations that required pretest counseling or specific consent to HIV testing and, if so, whether the laws specified the content of the pretest counseling or informed consent. To better understand these laws in context, we first provide background on existing HIV testing policies.

### The development of special HIV testing procedures

In 1985, when a test for HIV first became available, there was little incentive for patients to be tested. The risks of being identified as HIV-infected were serious, with people with AIDS being discriminated against in housing, employment, insurance, and medical care. Some even called for quarantining all those with AIDS. Because there were no effective treatments for AIDS, the personal benefits of testing were limited to planning for one's own future and taking steps to avoid transmission to others [2]–[4].

Nevertheless, public health officials sought to encourage HIV testing with the expectation that if people knew their status, they might alter their behavior. Those who received a negative test might be more motivated to take precautions to avoid infection. Those who received a positive test might take steps to avoid transmitting the virus. To encourage testing, special procedures were recommended to protect those who tested. Although physicians have a legal and ethical obligation to obtain consent for medical tests and treatment, in practice many medical interventions are undertaken with verbal consent and may not be specific [29]. For HIV, it was recommended that testing should be conducted only with specific consent, preferably in writing. In addition, testing should occur only after counseling; additional counseling should accompany disclosure of test results. HIV test results should receive special confidentiality protections beyond those ordinarily given to medical information. People could also test anonymously at separate test sites. Many states codified these procedures into law [2]–[4].

## Methods

We identified relevant statutes and regulations using electronic legal databases. We searched for laws in each state that addressed HIV testing in medical care. Search terms included “human immunodeficiency,” “HIV,” “acquired immunodeficiency,” “AIDS,” and “test” and its variants. In particular, we searched for amendments to HIV testing laws that were subject to our previous report [28]. We analyzed the content of statutes according to their plain meaning, in accordance with legal standards.

## Results

Our analysis of state laws regarding HIV testing demonstrates that the policies adopted early in the epidemic to encourage testing mostly remain in place today. We summarize relevant state requirements for HIV testing as follows:

### Requirements for informed consent and pre-test counselling

We found that the majority of states have laws that require specific consent for HIV testing. Fourteen states require written informed consent. Nineteen states permit oral consent, though 5 of these require consent to be documented. Eleven states require pretest counseling–2 of which require counseling take place face-to-face.

### Information disclosure

We found that 24 states require disclosure of specific information during pretest counseling and/or the informed consent process. Three of these states also recommend that additional information be disclosed with the required disclosures. States vary considerably in the amount of information they require to be disclosed, ranging from 1 required topic (4 states) to 9 required topics (3 states), with an average of approximately 5 topics. Table 1 summarizes the topics that states require or recommend be disclosed during pretest counseling and/or informed consent before HIV testing. Only one state specifies that such disclosures are not required for people who have previously tested and decline the information [30].

**Table 1:** Information to be disclosed in pretest counseling and/or informed consent for HIV testing

Topic	No. states require topic	No. states recommend topic
The nature of the HIV test	20	1
HIV risk behaviors and prevention measures	12	1
Confidentiality/exceptions to confidentiality of HIV test	12	0
That testing is voluntary	11	1
That anonymous testing is available	10	0
The nature of HIV/AIDS	8	2
Right to withdraw consent for HIV testing	8	1
The risks/benefits of HIV testing	7	1
The availability of referrals for information about or treatment for HIV	4	0
How HIV test results may be used	3	0
How HIV testing may affect the ability to obtain services	1	0

Only one state that amended its statutes since 2004 made changes to facilitate implementation of routine testing, and that was for prenatal testing, where there have been strong recommendations to do so since 1998 [31]–[33]. The remainder retained existing requirements. For example, one state that amended its prenatal testing laws retained requirements for pretest counseling, including disclosure of specific information [34]. Another state that amended its prenatal HIV testing law continues to require written consent, despite endorsing the IOM's recommendations for routine testing with notification and opt-out [35]. We found similar trends in four states that changed their general HIV testing laws. One of these was introduced and adopted after the CDC's recommendations were reported. The amended statute ties state recommendations on HIV testing to the CDC's “most current guidelines,” but it *explicitly rejects* essential components of the recommendations, stating that the CDC's guidelines “shall in no event be interpreted or implemented in a manner inconsistent with the minimum informed consent standards of this [statute] [36].” Two of the other states changed their laws to require *more* information to be conveyed during the consent process than they previously required [37], [38].

## Discussion

There is general agreement that we must increase HIV testing and decrease the number of people who are unaware they are HIV-infected. The CDC recommends routine HIV testing as one way to achieve this goal. Although its recommendations may be influential, the CDC does not have the authority to impose them on the states. The Constitution grants certain powers to the federal government, and all other powers are reserved to the states [39]. The regulation of health and public health are commonly recognized as state issues [40]. Accordingly, states hold the ultimate authority for HIV testing policy.

We found significant legal barriers to implementing the CDC's recommendations for routine HIV testing. Because of state requirements for specific consent to HIV testing, written consent to testing, and disclosure of specific information during pretest counseling or the informed consent process, the majority of states would need to amend their laws to permit routine HIV testing. For example, states that require disclosures would need either to eliminate those disclosures or make them recommendations, rather than requirements. However, our findings show that legislatures have not made the legal changes necessary to facilitate more routine HIV testing, despite strong public health recommendations to do so. In fact, the trend in states that have amended their laws since 2004 has been to reaffirm requirements for pretest counseling and consent, even, in some instances, while acknowledging the recommendations for more routine testing.

Even states without HIV testing statutes may face legal barriers to implementing the CDC's recommendations for routine HIV testing. Based on case law, many states use a “reasonable patient” standard for informed consent to medical treatment; physicians must disclose what a patient with ordinary reason and intelligence would want to know in making a medical decision. Because policy has recommended specific consent to HIV testing after pretest counseling and due to continued stigma surrounding HIV, it is likely that the “reasonable patient” standard would require more information about HIV testing than is currently contemplated under the CDC's recommendations. It may take time–and education efforts–to change public perceptions and to make routine testing acceptable.

Understanding these legal barriers and the apparent resistance to changing state HIV testing policies is essential to effecting any policy change that might increase HIV testing. The legislative history is sparse, but press reports suggest that the concerns that led to the adoption of special HIV testing legislation still resonate today. For example, legislators and HIV advocates have expressed concerns that the very limited disclosures that the CDC recommends are insufficient for making informed decisions about testing [22], [23], [41]. They suggest that additional procedures are still necessary for HIV testing because of the potential serious and negative effects, such as stigma and discrimination, that may be associated with HIV testing and infection [21], [23], [41], [42]. Some contend that the risks are greater because states now require names-based HIV reporting [21]. There is some evidence to support these concerns.

While the risks and benefits of testing have changed substantially with the advent of antiretroviral treatment [4], people living with HIV are still victims of discrimination, violence, and other social harms. Twenty to twenty-five percent of people living with HIV report experiencing discrimination in medical care and employment [43]–[47]. A study of discrimination claims filed with the Equal Employment Opportunity Commission (EEOC) supports these self-reports, finding HIV/AIDS-related employment discrimination to be “the most pervasive in terms of the number and magnitude of differences” and “particularly prevalent and conspicuous” compared to “a general disability population” [44]. In populations that already are subject to stigma or discrimination, including men who have sex with men and ethnic minorities, the risks of HIV testing may be higher [44]. Appreciating these psychosocial risks may be essential to making an informed decision about testing.

Eliminating pretest disclosures and counseling may also remove an important mechanism for educating individuals about HIV and reducing risk [21], [48]. Prevention remains the best strategy against HIV. Research has previously demonstrated that counseling is effective in helping people change their behaviors to prevent transmission of HIV [49], [50]; moreover, even brief, client-centered risk reduction counseling reduced both HIV risk behavior [49] and STD incidence [49], [51] compared to didactic informational prevention messages. Given that a major rationale for the CDC's revised recommendations is to detect HIV infection in individuals who either have never tested before, or who avoid retesting despite engaging in risk behavior, the need for pretest disclosures and counseling may be more important than ever, at least in the short term.

Another potential unintended consequence of these recommendations is that they may weaken protections, without substantially increasing testing among those who are unaware they are HIV-infected. The voluntary testing literature suggests access to a regular health care provider (HCP) and possession of health insurance predict test-seeking [7], [52]–[58]. Therefore, detection of HIV among those without a regular HCP or insurance may continue to prove challenging even if routine testing were implemented. In addition, a pattern of delayed or avoided testing among lower-income, less-educated, and ethnic minority populations is also found in the literature [7], [9], [56], [59]. It is possible that lack of access to HCP and health insurance, as well as inaccurate perceptions of individual- and community-level risk for HIV infection may be concentrated at this end of the socioeconomic spectrum. Finally, increasing the number of individuals in this sub-population who know their HIV-positive status may only serve to increase their social vulnerability. Specific interventions that provide support to socially vulnerable HIV-positive individuals may be required before this policy recommendation can be fully implemented.

These are important considerations, and developing a deeper understanding of them may suggest policy alternatives that may be more broadly acceptable, while achieving the important public health goal of increasing HIV testing. For example, written consent may be beneficial as a reminder to clinicians of their obligations to obtain specific consent to HIV testing and as a deterrent to unconsented testing. But documenting consent in the medical chart might be a viable alternative to written consent [48]. Similarly, it may be that not all of the information disclosed during pretest counseling and the informed consent process is essential to sound decision-making or that some of it could be conveyed in writing, rather than orally [42]. Flexibility in requirements may allow procedures to be appropriately tailored–for example, to distinguish between people testing for the first time and those who are repeat testers.

Successful examples of implementing more routine testing without abandoning specific consent exist. For example, the emergency department (ED) at Highland Hospital, an urban, academic teaching hospital in the San Francisco Bay Area, studied the feasibility of routinely offering HIV testing to patients. It used a combined approach of providing written information, using posters about HIV testing in the ED and informational brochures to answer basic questions about HIV, and clinician interactions. Triage nurses ask patients whether they are interested in testing, using a specially designed consent form and a script. Similarly, the Adolescent AIDS Program at Children's Hospital at Montefiore Medical Center in New York sought to make HIV testing more routine in heath care services. They developed a protocol to help providers increase testing, which includes a pocket guide for providers, a poster promoting routine HIV testing, patient HIV education brochures, and a substantial shortening of the time for pre-test counseling. A randomized trial of the protocol in community health clinics showed a doubling of testing among the intervention sites [24].

More public discussion of the CDC's recommendations is needed. Although the CDC sought public input into its recommendations, some HIV/AIDS advocacy organizations complained that the process was inadequate [20], [22]. Different stakeholders–public health and medical professionals, HIV advocates, and legislators – come to the debate with different perspectives. These perspectives must be taken into account if we are to develop an HIV testing policy that is generally acceptable and implementable. Moreover, many organizations already are looking at how to implement the recommendations in the clinical setting and elsewhere [24], [25], [27]. One of these reports an increase in testing under new procedures [27]. However, these organizations may not understand how state laws may limit their ability to implement the CDC recommendations. Potentially, these organizations could be in legal jeopardy for following the CDC's recommendations, but violating state law.

In sum, the CDC's recommendations for routine HIV testing represent an important change in the public health approach to HIV. However, states are ultimately responsible for implementing HIV testing policy, and individual states may have different concerns. More attention needs to be focused on understanding why states appear to have been reluctant to adopt HIV testing policies that permit more routine testing and to develop policy options that will be acceptable to them.

## References

[1] Branson BM, Handsfield HH, Lampe MA, Janssen RS, Taylor AW (2006). Revised Recommendations for HIV Testing of Adults, Adolescents, and Pregnant Women in Health-Care Settings.. MMWR.

[2] Centers for Disease Control and Prevention (2001). Revised guidelines for HIV counseling, testing and referral.. MMWR.

[3] Bayer R, Fairchild AL (2006). Changing the paradigm for HIV testing–the end of exceptionalism.. N Engl J Med.

[4] Gostin LO (2006). HIV Screening in Health Care Settings: Public Health and Civil Liberties in Conflict?. JAMA.

[5] Sanders GD, Bayoumi AM, Sundaram V, Bilir SP, Neukermans CP (2005). Cost-effectiveness of screening for HIV in the era of highly active antiretroviral therapy.. N Engl J Med.

[6] Mimiaga MJ, Goldhammer H, Belanoff C, Tetu AM, Mayer KH (2007). Men Who Have Sex With Men: Perceptions About Sexual Risk, HIV and Sexually Transmitted Disease Testing, and Provider Communication.. Sex Transm Dis.

[7] Lopez-Quintero C, Shtarkshall R, Neumark YD (2005). Barriers to HIV-testing among Hispanics in the United States: analysis of the National Health Interview Survey, 2000.. AIDS Patient Care STDS.

[8] Kaiser Family Foundation (2006). http://www.kff.org/kaiserpolls/upload/7521.pdf.

[9] Inungu JN (2002). Potential barriers to seeking human immunodeficiency virus testing among adults in the United States: data from the 1998 National Health Interview Survey.. AIDS Patient Care STDS.

[10] Freedberg KA, Samet JH (1999). Think HIV: why physicians should lower their threshold for HIV testing.. Arch Intern Med.

[11] Centers for Disease Control and Prevention (1999). Counseling Protocols, Standard Test Session 1 and 2 Counseling Protocol.. http://www.cdc.gov/hiv/projects/respect-2/counseling.htm.

[12] Centers for Disease Control and Prevention (1999). Counseling Protocols, Rapid Test Session Counseling Protocol.. http://www.cdc.gov/hiv/projects/respect-2/counseling.htm.

[13] Gallant JE (2004). HIV counseling, testing, and referral.. Am Fam Physician.

[14] Paltiel AD, Weinstein MC, Kimmel AD, Seage GR, Losina E (2005). Expanded Screening for HIV in the United States–An Analysis of Cost-Effectiveness.. N Engl J Med.

[15] Chase M (2006). CDC to Urge Routine HIV Tests for a Broad Swath of Americans. Wall Street Journal..

[16] Levine S (2006). D.C. Wants HIV Testing for All Residents 14 to 84. The Washington Post..

[17] Szymanski Z (2006). Officials move to block HIV testing changes. Bay Area Reporter. San Francisco.. http://www.ebar.com/common/inc/article_print.phpsecnewsarticle855.

[18] American Civil Liberties Union (2006). HIV Activists Tell New York Department of Health to Stop Gutting Informed Consent Laws. New York City.. http://www.aclu.org/hiv/privacy/25557prs20060515.html.

[19] Graham J, Kotulak R (2006). Call for routine HIV testing gets mixed response. Chicago Tribune. Chicago..

[20] (2006). Federal HIV Testing Initiative Can Only Succeed with Expanded Healthcare, Patient, and Provider Education, from 34 regional and national organizations.. http://www.champnetwork.org/media/Testing-Statement-092106.pdf.

[21] (2006). ACLU Says New CDC HIV Testing Recommendations Raise Health and Civil Liberties Concerns.. http://www.acul.org/hiv/testing/26819prs20060921.html.

[22] (2006). Letter to Division of HIV/AIDS Prevention, National Center for HIV, STD and TB Prevention, Centers for Disease Control and Prevention, Re: Comments on CDC Revised HIV Testing Guidelines from 54 community based organizations.. http://www.champnetwork.org/media/Testing-Letter.pdf.

[23] Chan S (2006). Rifts Emerge on Push to End Written Consent for HIV Tests. New York Times..

[24] (2006). Advisory Panel Meeting on the Implementation of the 2006 CDC Revised Recommendations for HIV Testing of Adults, Adolescents, and Pregnant Women in Health Care Settings, October 16, 2006, Atlanta, Georgia, Meeting Summary.. http://www.aahivm.org/index.phpoptioncom_contenttaskviewid492Itemid128.

[25] AIDS Education and Training Centers National Resource Center (2007). AETC Responses to 2006 CDC HIV Testing Recommendations: Resources.. http://www.aidsetc.org/aidsetcpageet-aetc-testing.

[26] Nielsen N (2006). AMA welcomes new Centers for Disease Control and Prevention (CDC) recommendations for routine HIV testing.. http://www.ama-assn.org/ama/pub/category/16871.html.

[27] Zelota NM, Klausner JD, Haller B, Nassos P, Katz MH (2007). Association Between Rates of HIV Testing and Elimination of Written Consents in San Francisco.. JAMA.

[28] Wolf LE, Lo B, Gostin LO (2004). Legal barriers to implementing recommendations for universal, routine prenatal HIV testing.. J Law Med Ethics.

[29] Beauchamp T, Childress J (2001). Principles of Biomedical Ethics. 2001: Oxford University Press..

[30] (2006).

[31] American Academy of Pediatrics, American College of Obstetricians and Gynecologists (1999). Joint statement of the American Academy of Pediatrics and the American College of Obstetricians and Gynecologists.. Pediatrics.

[32] Stoto M, Almario D, McCormick M (1999). Reducing the Odds: Preventing perinatal transmission of HIV in the United States..

[33] Centers for Disease Control and Prevention (2001). Revised recommendations for HIV screening of pregnant women.. MMWR Recomm Rep.

[34] (2006).

[35] (2006).

[36] (2006).

[37] (2006).

[38] (2006).

[39] Tribe LH (2000). American Constitutional Law..

[40] (2006).

[41] Freyer FJ (2006). Area doctors push for more HIV testing. The Providence Journal..

[42] San Francisco AIDS Foundation (2006). Comments on CDC's Revised Recommendations for HIV Testing of Adults, Adolescents, and Pregnant Women in Health Care Settings.. http://www.sfaf.org/policy/testing/cdc_guidelines.html.

[43] Schuster MA, Collins R, Cunningham WE, Morton SC, Zierler S (2005). Perceived discrimination in clinical care in a nationally representative sample of HIV-infected adults receiving health care.. J Gen Intern Med.

[44] Conyers L, Boomer K, McMahon BT (2005). Workplace discrimination and HIV/AIDS: The national EEOC ADA research project.. Work.

[45] Brooks RA, Etzel MA, Hinojos E, Henry CL, Perez M (2005). Preventing HIV among Latino and African American gay and bisexual men in a context of HIV-related stigma, discrimination, and homophobia: perspectives of providers.. AIDS Patient Care STDS.

[46] (1998).

[47] Huebner DM, Rebchook GM, Kegeles SM (2004). Experiences of harassment, discrimination, and physical violence among young gay and bisexual men.. Am J Public Health.

[48] The AIDS Institute (2006). http://www.theaidsinstitute.org/downloads/taicdctestingcomments.pdf.

[49] Kamb ML, Fishbein M, Douglas JM, Rhodes F, Rogers J (1998). Efficacy of risk-reduction counseling to prevent human immunodeficiency virus and sexually transmitted diseases: a randomized controlled trial. Project RESPECT Study Group.. JAMA.

[50] Weinhardt LS, Carey MP (2000). Human immunodeficiency virus testing and behavior change.. Arch Intern Med.

[51] Bolu OO, Lindsey C, Kamb ML, Kent C, Zenilman J (2004). Is HIV/sexually transmitted disease prevention counseling effective among vulnerable populations? a subset analysis of data collected for a randomized, controlled trial evaluating counseling efficacy (Project RESPECT).. Sex Transm Dis.

[52] Bond L, Lauby J, Batson H (2005). HIV testing and the role of individual- and structural-level barriers and facilitiators.. AIDS Care.

[53] Fernandez M, Perrino T, Royal S, Ghany D, Bowen G (2002). To test or not to test: are Hispanic men at highest risk for HIV getting tested?. AIDS Care.

[54] Herndon B, Asch SM, Kilbourne AM, Wang M, Lee M (2003). Prevalence and predictors of HIV testing among a probability sample of homeless women in Los Angeles County.. Public Health Rep.

[55] Tobin K, Tang A, Gilbert S, Latkin C (2004). Correlates of HIV antibody testing among a sample of injection drug users: the role of social and contextual factors.. AIDS Behav.

[56] Do TD, Chen S, McFarland W, Secura GM, Behel SK (2005). HIV testing patterns and unrecognized HIV infection among young Asian and Pacific Islander men who have sex with men in San Francisco.. AIDS Educ Prev.

[57] Do TD, Hudes ES, Proctor K, Han CS, Choi KH (2006). HIV testing trends and correlates among young Asian and Pacific Islander men who have sex with men in two U.S. cities.. AIDS Educ Prev.

[58] MacKellar DA, Valleroy LA, Anderson JE, Behel S, Secura GM (2006). Recent HIV testing among young men who have sex with men: correlates, contexts, and HIV seroconversion.. Sex Transm Dis.

[59] Johnson DF, Sorvillo FJ, Wohl AR, Bunch G, Harawa NT (2003). Frequent failed early HIV detection in a high prevalence area: implications for prevention.. AIDS Patient Care STDS.

